# Microfluidic droplet enrichment for targeted sequencing

**DOI:** 10.1093/nar/gkv297

**Published:** 2015-04-14

**Authors:** Dennis J. Eastburn, Yong Huang, Maurizio Pellegrino, Adam Sciambi, Louis J. Ptáček, Adam R. Abate

**Affiliations:** 1 Department of Bioengineering and Therapeutic Sciences, California Institute for Quantitative Biosciences, University of California, San Francisco, CA 94158, USA; 2 Mission Bio, Inc., San Francisco, CA 94107, USA; 3 Department of Neurology and Howard Hughes Medical Institute, University of California, San Francisco, CA 94158, USA

## Abstract

Targeted sequence enrichment enables better identification of genetic variation by providing increased sequencing coverage for genomic regions of interest. Here, we report the development of a new target enrichment technology that is highly differentiated from other approaches currently in use. Our method, MESA (Microfluidic droplet Enrichment for Sequence Analysis), isolates genomic DNA fragments in microfluidic droplets and performs TaqMan PCR reactions to identify droplets containing a desired target sequence. The TaqMan positive droplets are subsequently recovered via dielectrophoretic sorting, and the TaqMan amplicons are removed enzymatically prior to sequencing. We demonstrated the utility of this approach by generating an average 31.6-fold sequence enrichment across 250 kb of targeted genomic DNA from five unique genomic loci. Significantly, this enrichment enabled a more comprehensive identification of genetic polymorphisms within the targeted loci. MESA requires low amounts of input DNA, minimal prior locus sequence information and enriches the target region without PCR bias or artifacts. These features make it well suited for the study of genetic variation in a number of research and diagnostic applications.

## INTRODUCTION

Next-generation sequencing technologies have brought about a steep decline in the per-nucleotide cost of sequencing. Nonetheless, the cost of sequencing larger eukaryotic genomes is still substantial and the quantity of sequence data generated can be complicated to process and analyze (1). Beyond the expense and complexity of whole-genome sequencing, for many studies, this sequence information is superfluous to the goals of the study. These studies are often focused on analyzing only a subset of protein-coding or regulatory regions of the genome thought to harbor genetic alterations linked to a specific disease or phenotype. Additionally, association studies of genetic disorders often require thousands of samples to achieve statistical power making whole-genome sequencing unfeasible. To address these issues, several methods have been developed to selectively enrich for genomic regions of interest (2,3). These methods enable researchers to focus the entire capacity of the sequencing instrument on the region of interest, reducing cost, simplifying data analysis and yielding higher sequence coverage for the desired region.

The most common target enrichment methods rely on hybridization-based capture (either in-solution or on-array) or highly multiplexed polymerase chain reaction (PCR) (3). Hybridization capture is most effective for enriching large genomic regions; however, this method requires a considerable amount of input DNA and can often produce results that lack the uniformity and specificity of sequence coverage desired for the target region (4–12). Multiplex PCR methods provide high specificity target enrichment, but are difficult to implement and scale for the enrichment of hundreds of kilobases (13–16). Current target enrichment methods can also be laborious and expensive, requiring the design and synthesis of thousands of primer sets or hybridization probes. Therefore, new approaches are needed to overcome the limitations of the current technologies and make targeted enrichment more widely useful for the analysis of genetic variation.

Droplet-based microfluidic methods are well suited for a variety of ultrahigh-throughput molecular biology applications (17–19). These methods rely on picoliter-volume droplets of aqueous biological reagents encapsulated in an oil-based emulsion (20). The microdroplets can enable massively parallel PCR reaction capability on purified nucleic acids or single cells with minimal time and reagent cost (21–24). When combined with microfluidic sorting techniques, single-cell TaqMan-based PCR reactions performed in droplets can be used to trigger sorting and isolate specific cells of interest at ultrahigh-throughput levels (22).

In this study, we developed and characterized a new microfluidic technology for sequence enrichment that does not rely on hybridization-based capture or PCR amplification to perform target enrichment. Instead, our approach physically encapsulates, without bias, diluted nucleic acids into millions of microdroplets where the target sequence is unambiguously identified with TaqMan PCR. Once identified, microfluidic sorting enables isolation and enrichment of the target nucleic acid for downstream sequencing. This novel approach to targeted sequence enrichment addresses many of the shortcomings of current methods.

## MATERIALS AND METHODS

### Cell culture and DNA isolation

Patient derived lymphoblast cells were cultured at 37°C with 5% CO_2_ in Roswell Park Memorial Institute (RPMI) 1640 supplemented with 15% fetal bovine serum (FBS) and L-Glycine. The cells were grown to ∼800 000 cells/ml. To arrest the cells in metaphase, colcemid was added to a final concentration of 0. 1 μg/ml and the cells were cultured for another 4 h before harvest. The cells were then washed twice with pre-chilled phosphate buffered saline (PBS) and re-suspended in warm PBS. The cells were then mixed with equal volume pre-warmed 2% SeaPrep LMP agarose solution, which has a gelling point of ∼20°C and a melting point of ∼50°C. The mixture was poured into PCR tube molds and cooled on ice to gel. In this way, the cells were embedded and immobilized in the gel plugs. To isolate cellular genomic DNA, the gel plugs were treated with lysis buffer (0.5 M ethylenediaminetetraacetic acid, pH 9.4, 1% wt/vol sodium lauryl sarcosine and 0.3 mg/ml proteinase K) at 37°C for 24 h (37°C was used so that the gel plugs were not melted). The gel plugs were further washed with ice-cold Tris-EDTA (TE) containing 0.1 mM phenylmethanesulfonyl fluoride (PMSF) to remove proteinase K. The resulting gel plugs contained largely intact genomic DNA and were stored at 4°C (25). A small portion of the plugs was reserved for quantification using the PicoGreen dsDNA assay kit from Life Technologies.

Prior to use in the PCR reactions, an appropriate volume of the SeaPrep gel plug was cut and melted at 55°C for 2–3 min in a thermocycler. Although a small number of gel fragments sometimes remained in the DNA solution, we found they had little, if any, effect on the preparation of the PCR reactions. A pipet was used to transfer the DNA into the final reactions prior to droplet encapsulation.

### Fabrication and operation of microfluidic devices

The microfluidic devices were fabricated from polydimethylsiloxane molds that were created by soft lithography and subsequently bonded to glass and made hydrophobic with Aquapel (26). Flow-driven and computer-controlled syringe pumps (NewEra) injected reagents through polyethylene tubing into the fabricated devices. DNA to be enriched was added to PCR reactions and encapsulated in droplets dispersed in fluorinated oil (Novec 7500) containing a 5% stabilizing, PEG-PFPE block-copolymer surfactant (27).

### Microdroplet TaqMan reverse transcriptase-PCR

TaqMan assay primers for the NFAT5-targeted region were as follows: Primer1 5′-GTT TCC TCA CTT CAG AAC CCA–3′, Primer2 5′-GTG ACC CTT GTA CCA ACT GAA-3′, NFAT5 TaqMan probe 5′-/56-FAM/CAC AGA CCC/ZEN/CCT TGT TCC ATA GCT/3IABkFQ/-3′. TaqMan assay primers for the COG4-targeted region were as follows: Primer1 5′-GCC CTC AAA CAC TAC AGC AA-3′, Primer2 5′-CAG CCT CTC CTC AAA GTC AA-3′, COG4 TaqMan probe 5 5′-/56-FAM/TAC CCC TGC/ZEN/CAG TCC TAG ACA ACT/3IABkFQ/-3′. TaqMan assay primers for CLEC18A,B,C-targeted regions were as follows: Primer1 5′-CCC ACC TAT TCC CTT TCC TG-3′, Primer2 5′-GAG TCT GGT CTT CCT CTT CTT G-3′, CLEC18 TaqMan probe 5′-/56-FAM/ACC CAG AAC/ZEN/TTC CAG AGA GTG TCC T/3IABkFQ/-3′. All TaqMan reaction primers and probes were purchased as a pre-mixed assays from Integrated DNA Technologies (IDT). AmpliTaq Gold DNA polymerase mix (Applied Biosystems) was used for the microdroplet TaqMan PCR reactions. Hundred nanogram of purified genomic DNA was used for each emulsified PCR reaction. Thermocycling conditions were 93°C for 2 min followed by 45 cycles of: 92°C, 15 s, 56°C, 10 s and 60°C, 50 s.

### Ultrahigh-throughput sorting of droplets

Thermocycled droplets were transferred to a 1-ml syringe and reinjected into the microfluidic droplet sorter. Droplets were spaced with carrier oil with a lower 0.25% surfactant concentration. The droplet fluorescence was excited with a 473-nm laser (CNI lasers) and measured with a photomultiplier tube (Thorlabs). An FPGA card (National Instruments) running custom LabVIEW code interpreted the fluorescence signal in real time, sorting droplets falling within user-defined ranges of fluorescence amplitude and size (temporal width). The dielectrophoretic sorting pulse output by the FPGA was amplified with a high-voltage amplifier (Trek) to 1 kV and applied to an on-chip salt water electrode (28).

### DNA recovery and sequencing

Positively sorted TaqMan emulsions were broken using perfluoro-1-octanol and the aqueous fraction was diluted in 10 mM Tris pH 8.0. The aqueous fraction containing the enriched genomic DNA was then treated with uracil-DNA glycosylase (UDG) (New England Biolabs) for 2 h at 37°C to prevent carry over TaqMan PCR amplicons in the downstream sequencing library preparation steps. Glycosylase reactions were subsequently ethanol precipitated to purify and concentrate the DNA prior to whole genome amplification (WGA). WGA was performed using a REPLI-g mini kit from Qiagen that relies on Multiple Displacement Amplification (MDA) technology. Fifty percent of the MESA (Microfluidic droplet Enrichment for Sequence Analysis) sorted genomic DNA from eight enrichment experiments was amplified with this method. Following WGA, the amplified DNA was purified using a QIAamp DNA mini kit (Qiagen).

For next-generation sequencing, 1 ng of REPLI-g-amplified DNA was subsequently used for sequencing library preparation with a Nextera XT library kit (Illumina). Six replicate libraries were prepared from the pooled DNA sample and appropriately barcode indexed for partitioning during sequence data analysis. For the control reference genome, 1 ng of pre-enriched genomic DNA was used directly as input for Nextera XT library preparation. Quality and size distributions of all libraries were analyzed using a High Sensitivity DNA Assay run on a Bioanalyzer (Agilent). Sequencing was performed with a HiSeq2500 sequencer with 50-bp reads. The six individually prepared MESA sequencing libraries were run on two lanes (three libraries per lane) of the HiSeq2500. Total sequencing reads obtained for the libraries ranged from 22 296 448 to 83 174 506 (Table 1). The pre-enriched genomic DNA reference library was run individually on a single lane of the HiSeq2500 and generated a total of 197 210 836 reads.

**Table 1.** Summary of data from MESA-generated sequencing libraries

MESA library #	1	2	3	4	5	6	Average ± SEM
Alignment rate	95.00% (20 899 959)	91.06% (20 032 446)	95.85% (21 086 229)	96.21% (21 165 491)	95.95% (21 109 421)	95.14% (20 930 295)	94.87% ± 0.79% (20 870 640 ± 172 956)
Uniquely aligned (MAPQ > 1)	85.19% (18 742 409)	81.32% (17 889 326)	85.70% (18 853 279)	85.27% (18 760 481)	85.72% (18 859 069)	85.03% (18 706 216)	84.71% ± 0.69% (18 613 674 ± 167 385)
More than 1 alignment	9.81% (2 157 550)	9.74% (2 143 120)	10.15% (2 232 950)	10.93% (2 405 010)	10.23% (2 250 352)	10.11% (2 224 079)	10.16% ± 0.17% (2 251 102 ± 38 949)
Failed to align	4.93% (1 084 091)	8.87% (1 951 375)	4.09% (899 010)	3.72% (819 242)	3.98% (876 035)	9.64% (2 120 956)	5.87% ± 1.09% (1 333 324 ± 263 341)
Duplicates	11.56% (2 416 086)	11.29% (2 260 699)	10.72% (2 261 210)	9.58% (2 028 249)	9.17% (1 935 529)	10.19% (2 133 638)	10.42% ± 0.39% (2 123 865 ± 58 558)
Total reads	22 296 448	27 687 547	83 174 506	52 175 999	52 015 474	44 830 563	47 030 090 ± 8 847 746

Percentages were calculated from the analysis of 22 million reads randomly sampled from each library. Read numbers are listed in parentheses.

### Sequence analysis of sorted genomic DNA

Reads passing quality control were aligned to the hg19 human genome using the Burrows–Wheeler Aligner (29). Coverage and pileup analysis were performed using samtools (30) and bedtools (31), then plotted using ggplot2 for R (32). The number of duplicates was calculated using picard tools (http://broadinstitute.github.io/picard/). For Figures 3 and 4, a total of 130 million reads were analyzed for the MESA-enriched samples (22 million reads each) and the non-enriched sample. The average coverage plots were generated using a sliding window of 20 kb with a 2-kb step. For Table 1, the number of unique and not unique alignments was extracted using the XT:A:U and XT:A:R flags, respectively.

**Figure 1.**
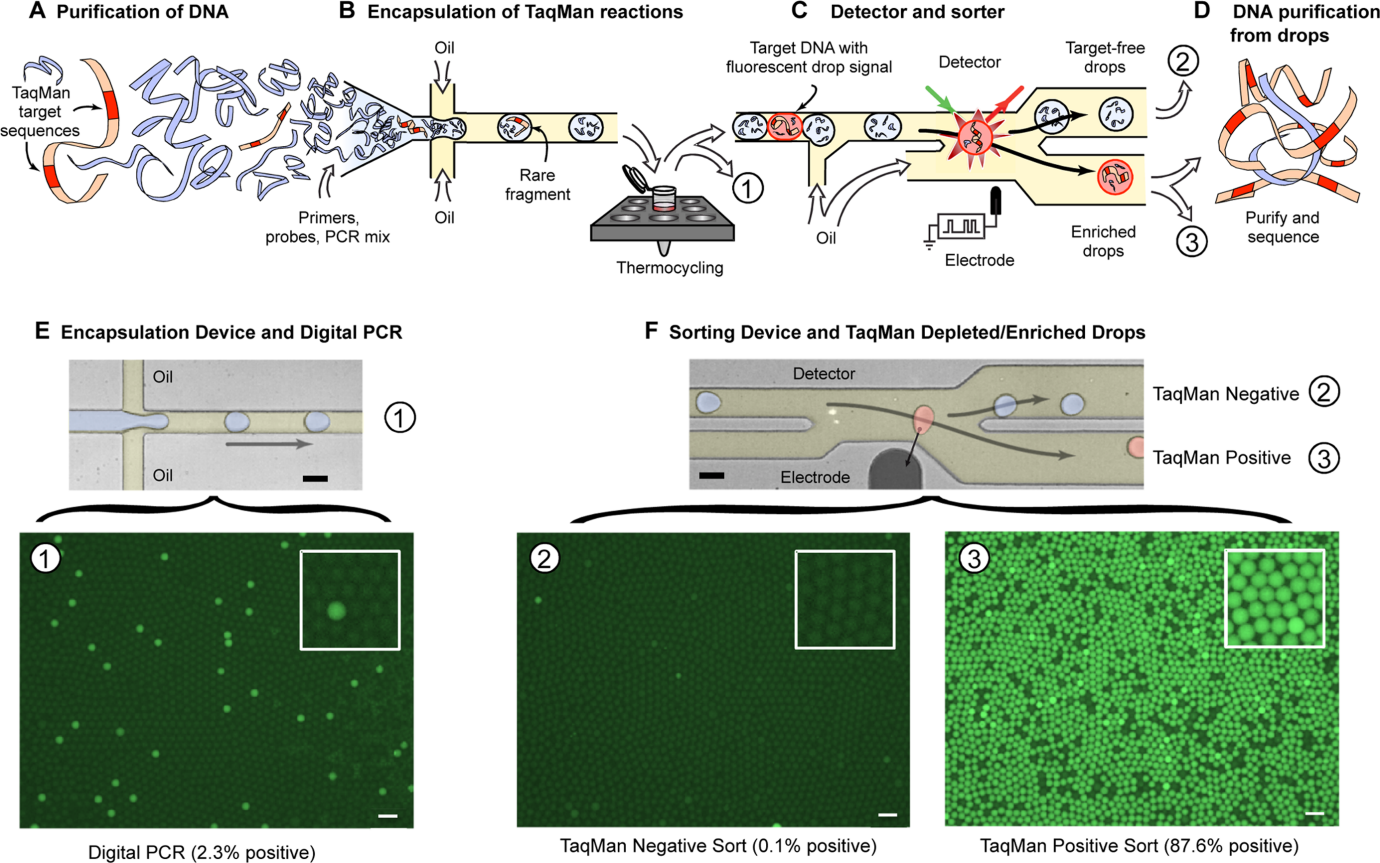
Workflow for microfluidic droplet-based target enrichment. (**A**) TaqMan assays are designed to target genomic DNA loci of interest for microfluidic enrichment. (**B**) PCR reagents, TaqMan primers/probes and genomic DNA are encapsulated into aqueous microdroplets in an oil-based emulsion using flow focusing. (**C**) Emulsified microdroplets are thermocycled before being reinjected into a microfluidic sorter. The microfluidic sorter interrogates the droplets for TaqMan probe fluorescence and sorts the PCR positive droplets for collection and recovery of genomic DNA. (**D**) Next-generation sequence analysis is performed on enriched DNA. (**E**) Pseudocolored microscope image of the PCR droplet generating microfluidic device (scale bar = 50 μm). The fluid colored blue is the aqueous TaqMan PCR reaction mix that is encapsulated. The fluorescent micrograph below this device shows a field of digital PCR droplets with 2.3% of the droplets positive for TaqMan reaction fluorescence (FAM dye shown in green). These positive fluorescent drops indicate the presence of the genomic target sequence to be enriched. (**F**) Micrograph of the microfluidic sorter device (scale bar = 50 μm). During microfluidic droplet sorting, the TaqMan positive drops, pseudocolored red, are pulled by an electrode into the lower droplet channel (numbered 3) and sent for collection. The TaqMan/target negative droplets, pseudocolored blue, flow into the upper channel (numbered 2) where they are discarded. Below the sorting device image are TaqMan positive droplets that have been enriched to 87.6% (3) from the original 2.3% (1) positive digital PCR droplets shown in (E). Correspondingly, the droplets lacking target DNA collected from channel number 2 are depleted for TaqMan positive droplets (2). Scale bars for fluorescent drop fields are 100 μm. Inset panels in fluorescent micrographs show magnified portions of the drop field.

**Figure 2.**
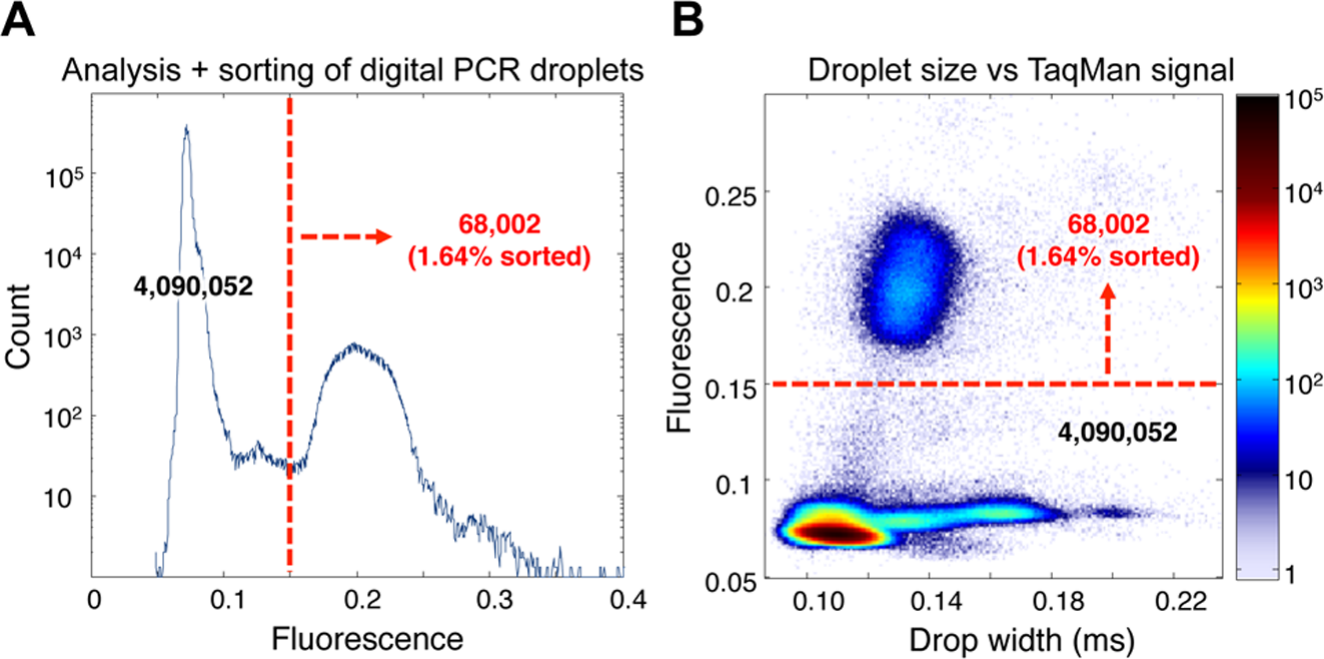
Digital PCR droplet detection and sorting data. (**A**) Analysis of FAM fluorescence from one of the replicate multiplex digital droplet PCR reactions reinjected into the microfluidic sorting device. (**B**) Scatterplot diagram of droplet size and FAM fluorescence values from the multiplex digital TaqMan reactions. The dashed red lines on the graphs indicate the fluorescence sorting threshold that was applied to identify 68 002 (1.64%) PCR-positive droplets for sorting and enrichment. Heat map indicates drop counts.

**Figure 3.**
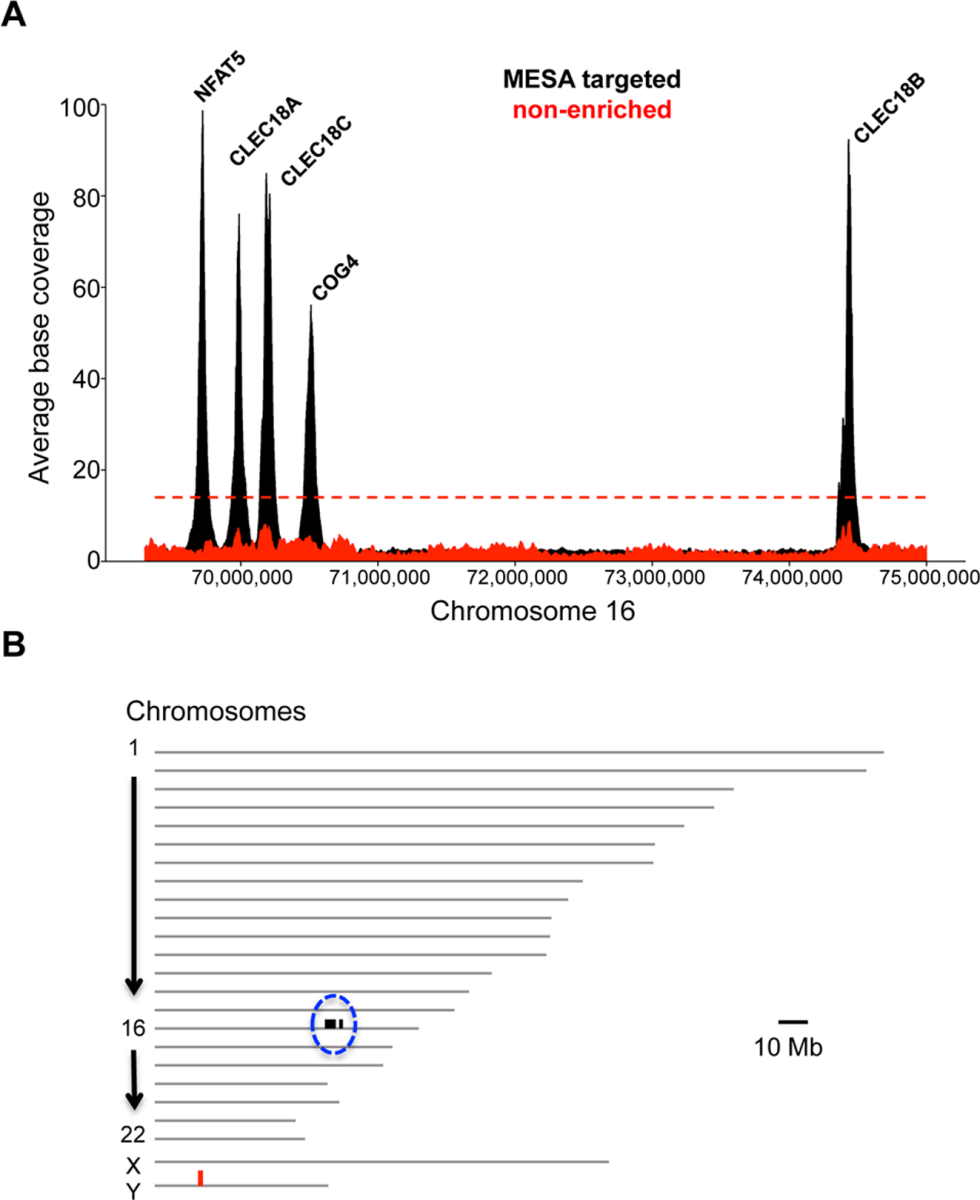
Specificity of MESA. (**A**) Average base coverage within the 6 Mb surrounding the targeted regions (NFAT5, CLEC18A, CLEC18C, COG4 and CLEC18B) on human chromosome 16 in the MESA-enriched (black) and non-enriched reference (red) samples. Average coverage calculated over a 20-kb sliding window. The dashed red line is drawn at a base coverage value of 13.62 and represents the mean plus three standard deviations from the average coverage for chromosome 16 in the non-enriched reference sample. (**B**) MESA enrichment is highly specific for the five TaqMan target regions on chromosome 16 (marked with black lines circled in blue), compared to the non-enriched reference genome sample (red lines). Only areas of enrichment exceeding a minimum coverage threshold of 56X and differentially present in either MESA or non-enriched samples are indicated with lines. Human chromosomes are depicted as gray lines and drawn roughly to scale.

**Figure 4.**
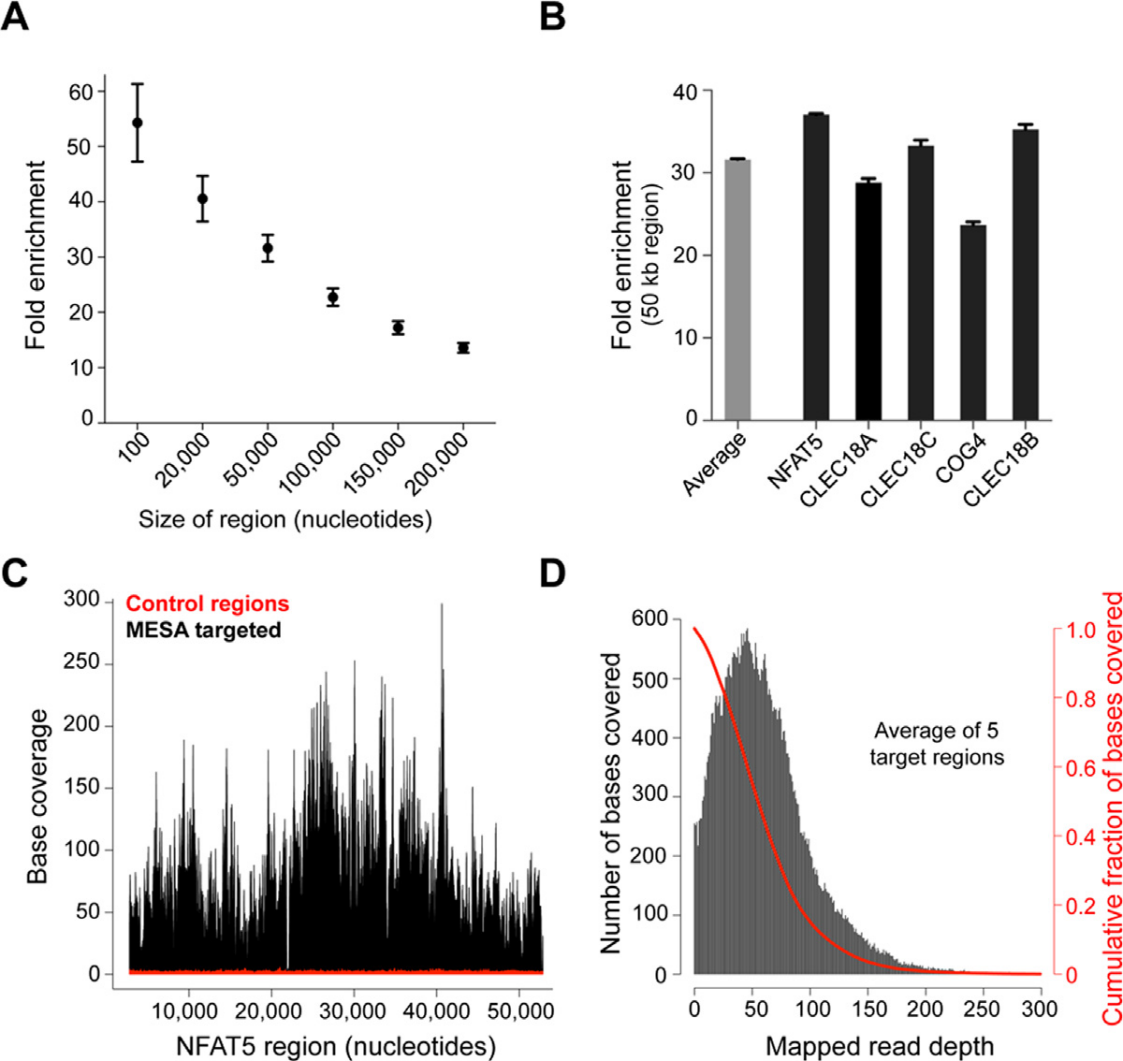
Characterization of target enrichment with next-generation sequencing. (**A**) Average fold sequence enrichment with MESA for various target region sizes ranging from 100 to 200 kb (centered on TaqMan assay target sites). The data show that the MESA approach can enrich even 200 kb genomic regions more than 13-fold using a single TaqMan assay. (**B**) The bar graph shows the fold enrichment of each targeted locus across a 50 kb region relative to the average coverage for the entire genome. The average enrichment of all five target regions is also shown. (**C**) Representative base coverage of the ∼50 kb region corresponding to the NFAT5 target locus (black), compared to the average coverage achieved within 10 non-targeted genomic regions of the same size (red). (**D**) Uniformity of coverage. The plot shows the distribution of bases covered at different depths across the five targeted 50-kb regions (black), and the cumulative fraction of bases with a coverage at or above the specified depth (red).

Bcftools was used for single nucleotide polymorphism (SNP) calling, disregarding duplicated reads and filtering out variants with a phred-scaled quality score and read depth lower than 10 (30). The SNPs were compared to SNPs identified by the 1000 genome project and submitted to the UCSC common SNPs database (Build 141) (33).

## RESULTS

### MESA workflow

Although microfluidic droplet-based PCR reactions have been used to amplify target molecules for sequencing (15), the MESA approach is unique in that the droplet PCR reactions are not used to directly amplify the material to be sequenced. Instead, MESA employs droplet-based TaqMan PCR solely as a means to identify droplets containing nucleic acid target regions. With this approach, a mixed population of nucleic acids such as purified genomic DNA fragments is first subdivided and compartmentalized into millions of discrete microdroplets. This subdivision results in the target nucleic acid occupying some of the droplets while others lack the target and contain only ‘background’ nucleic acid sequences to be discarded. The droplets containing the target sequence are then identified via TaqMan PCR-generated fluorescence and isolated with dielectrophoretic droplet sorting (22,26). The enrichment factor for the target sequence can be controlled by varying the target nucleic acid concentration in the PCR reaction. Diluting the DNA reduces the number of ‘background’ molecules co-encapsulated with the targets, thus yielding higher enrichments, but comes at the cost of reducing the number of targets recovered. For a maximum number of droplets, there is thus a tradeoff between the purity of the enriched fraction and the number of target molecules recovered. Following target enrichment, the relatively small TaqMan amplicons produced during the PCR reaction are enzymatically removed with UDG. The isolated target sequences are then purified and prepared for next-generation sequencing. The MESA workflow and requisite microfluidic devices are shown in Figure 1.

### Microfluidic enrichment of genomic DNA

TaqMan PCR affords single molecule sensitivity and high specificity making it ideal for accurately identifying droplets containing target nucleic acids (21,34). This high degree of specificity enables the accurate identification of target nucleic acids with minimal (∼100 bp) prior sequence information. Additionally, using multiplexed TaqMan assays, MESA enables the simultaneous enrichment of multiple target regions. To demonstrate this, we designed a multiplexed TaqMan assay targeting multiple genomic loci on human chromosome 16. Three TaqMan assays, NFAT5, CLEC18 and COG4, were designed to target five genomic sequence regions. Due to genomic sequence identity, the CLEC18 assay can amplify regions within the *CLEC18A, CLEC18B* and *CLEC18C* genes. All three TaqMan probes were labeled with fluorescein (FAM) fluorescent dye although, if desired, different colors can be used to identify which of these regions is present in a given droplet.

Eight replicate multiplex TaqMan digital droplet PCR reactions, each containing 100 ng of purified genomic DNA, were emulsified and thermocycled. Following thermocycling, the droplets were reinjected into the microfluidic sorting device and examined for FAM fluorescence. Droplets were analyzed and sorted at a rate of 1 kHz. The average target detection rate with the TaqMan probes in the digital droplet PCR reactions across the eight experimental replicates was 1.3% (±0.6). A representative scatterplot of one of these replicates is shown in Figure 2. The droplets containing target genomic DNA were clearly identifiable as a cluster of FAM positive droplets. Dielectrophoretic droplet sorting was configured to trigger the sorting and collection of all of the droplets in this cluster (dashed red line in Figure 2). Merged droplets due to emulsion instability accounted for ∼2% of the sorted droplets (Figure 2B). Genomic DNA from the sorted droplets was isolated, pooled and isothermally amplified prior to sequencing library preparation.

### Sequence quality and specificity with MESA target enrichment

Six replicate Nextera libraries generated from MESA-enriched target-DNA were sequenced. A sequencing data set from non-enriched genomic DNA was also generated to serve as a reference sample. To evaluate our method, we considered an equal number of reads per sample and analyzed the first 22 million reads of each. This produced a combined data set that contained 130 million reads, equivalent to the output of less than one lane from a standard Illumina sequencing run. The sequencing data generated from MESA-enriched DNA was of high complexity, with a minimal number of duplicate and unaligned reads, as shown in Table 1. Additional analysis of the target regions demonstrated an even distribution of reads that map to the sense and anti-sense strand of genomic DNA, suggesting that our method displays minimal strand bias.

Since MESA enriches for specific genomic DNA regions, we expect the targeted loci to be over-represented within the reads. Indeed, in the MESA-enriched samples, the five target regions have an average base read depth that ranges from 56X to 98X for the COG4 and NFAT5 regions, respectively (Figure 3A). Enhanced base coverage was also centered on the *CLEC18A, CLEC18B* and *CLEC18C* genes that were simultaneously identified with the single CLEC18 TaqMan assay. Unexpectedly, several areas of ‘enhanced’ coverage from the non-enriched reference genome control sample roughly coincided with the peaks observed in the MESA samples. To establish whether this coverage was significant, we calculated the mean and standard deviation of the average coverage values across the chromosome. None of the average coverage values in the region were above three standard deviations from the mean (Figure 3B, dashed red line), suggesting that the observed variation is not significantly different from background. A possible explanation for the background variation is trace contamination with MESA samples during library preparation. However, the lack of enhanced base coverage at the COG4 and NFAT5 target regions makes this explanation unlikely. A more plausible reason for the reference genome variation at the CLEC18A,B,C target regions is transposase bias due to Nextera sequencing library preparation (35–37).

To further assess the specificity of MESA, we calculated the average coverage across the whole human genome and searched for other regions that exceeded an average coverage of 56X that were differentially present in our MESA-enriched data set but absent from the non-enriched reference genome. Consistent with the extreme specificity of TaqMan assays, the targeted regions on chromosome 16 were the only loci displaying such enhanced coverage (Figure 3B). The absence of off-target enrichment in the MESA-processed samples is strong validation for the overall specificity and uniformity of the approach.

### Characterization of MESA target regions with sequence analysis

Hybridization capture and PCR-based target enrichment strategies frequently require the design of a probe or primer set for every 1000 bp of sequence to be enriched. The MESA approach has the potential to simplify the number of oligonucleotides that must be designed and used by enriching for genomic sequences flanking the TaqMan reaction site on the DNA fragments that are emulsified. To illustrate this, we examined sequence read coverage for the genomic region on chromosome 16 that we targeted. This analysis demonstrated that a 200-kb region, centered on each of the TaqMan assays, showed sequence enrichment that was 13.6-fold higher relative to the genome-wide average coverage (Figure 4A). MESA target coverage increased to an average 31.6-fold enrichment over a 50-kb region, with each of the five targets displaying between 24- and 37-fold increases within this window size (Figure 4B). A more detailed analysis of base coverage depth is shown for the 50-kb NFAT5 target region in Figure 4C.

The genomic interval enriched in MESA samples is likely to correlate with the size of the encapsulated DNA fragments in the droplet PCR reactions. Therefore, we used the full width at the half maximum value of the average coverage distribution for each targeted locus as a proxy for fragment size (Supplementary Figure S1). This analysis suggested that an average fragment size of 52 400 (±5706) bp was encapsulated in the droplets.

To further evaluate the efficacy of our method, we calculated the uniformity of coverage within the regions of interest. This is a useful metric employed to determine the amount of sequencing necessary to reach a desired threshold of coverage, and is especially important when analyzing SNPs. Several groups have attempted to calculate the depth of coverage needed to reproducibly call SNP variants in the human genome (38–41), with estimates ranging from ∼10X for homozygous to ∼30X for heterozygous variants. In MESA-enriched samples, ∼95% and ∼77% of target bases are covered at >10X and >30X coverage depth, respectively, demonstrating that our method should effectively detect most SNP variants (Figure 4D).

### Accurate variant calling with MESA

**Figure 5.**
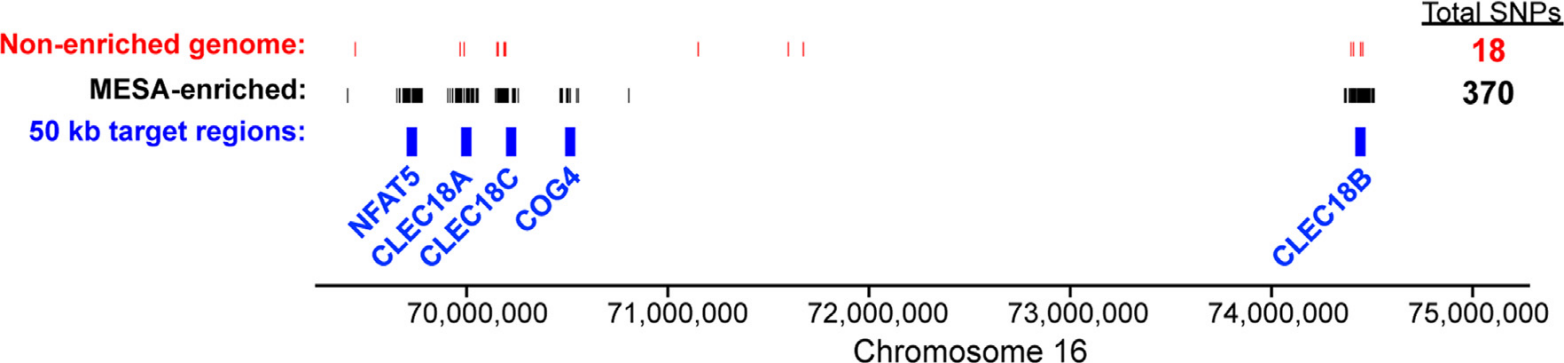
Accurate genetic variant calling with MESA. SNP calling across the region spanned by the targeted loci. SNPs identified in MESA-enriched samples (black) compared to non-enriched genomic DNA (red). The blue boxes represent the location of the 50 kb regions encompassing the TaqMan probes (blue).

To demonstrate the ability of MESA to improve SNP calling with low input DNA, we analyzed the genomic region containing the five targeted loci (∼6 Mb) and considered polymorphisms with both a minimum read depth and phred-scaled quality score of 10. In the MESA-enriched samples, we detected a total of 370 variant nucleotides, while only 18 were called in the non-enriched genomic sample (Figure 5). Equal numbers of reads (130 million) from MESA and non-enriched samples were used for the comparison. Since the regions immediately surrounding the probes have a much higher coverage than regions farther away, we expected to find more SNPs closer to the 50-kb-targeted regions. As predicted, we identified 116 SNPs (∼31% of the total SNPs found in the larger 6-Mb target region) within the probe-centered 50-kb regions in the MESA samples. In the non-enriched reference sample only four SNPs identified also fell within the 50-kb target region. Interestingly, almost all of the MESA-identified SNPs have been previously described (111/116, 96.5%) by the 1000 genome project (33), further corroborating the utility of MESA for variant analysis.

## DISCUSSION

Our technical advance has been to develop a robust microfluidic workflow that enables a completely new approach to targeted sequence enrichment. This method relies on a small number of TaqMan primer/probe sets that are easy to design and economical to purchase. The TaqMan reactions provide exquisitely specific detection of target sequences in our workflow, thus overcoming the specificity issues common to hybridization-based capture enrichment. Importantly, unlike other droplet-based PCR target enrichment methods, with MESA, the nucleic acids to be sequenced are not produced by PCR amplification (15). Rather ∼100 bp for each 50–200-kb-enriched target region is PCR amplified—for identification purposes only—and the resultant amplicons from this reaction are enzymatically removed from the sample prior to sequencing. The lack of direct PCR amplification not only enables enrichment of a larger target region without the need for multiplex primer sets or additional target sequence information but also aids uniformity of sequencing coverage by eliminating the error and bias common to many PCR-based enrichment methods.

MESA can simultaneously target and enrich multiple 50–200-kb loci. This capability is enabling for numerous applications such as targeted sequencing of a panel of tumor suppressors (6,14). However, for other applications, where larger genomic regions must be sequenced, it may be necessary to enrich a contiguous interval that spans several megabases. For these applications, multiple TaqMan probes can be targeted to produce overlapping regions of enrichment. Additionally, the current 200-kb-enriched target region is not likely the upper size limit for MESA. We estimated that our current DNA fragment size in the PCR droplets was ∼52 000 bp. Use of higher quality, large-fragment DNA in the initial PCR reaction as well as optimization of the microfluidic flow rates and droplet volumes should further increase the MESA enrichment interval. Higher dilution of target DNA molecules will also improve the enrichment at distal target region sites, effectively increasing the total target region size for use in subsequent sequence analysis.

A key feature of MESA is the scalability provided by the microfluidics. This scalability enables a large number of digital PCR droplets to be generated for a given concentration of target DNA. Correspondingly, fewer ‘off-target’ background DNA molecules are co-encapsulated in the same droplet as the target DNA, resulting in a higher overall enrichment factor achieved with the MESA workflow. In this study, droplets were processed at ∼1 kHz; however, with slight modifications, sorting devices have reached rates of 30 kHz using similarly sized droplets (42). MESA target enrichment can also be accomplished using traditional flow cytometers to sort double emulsions generated after the droplet PCR (43). The use of widely available flow cytometers for MESA has the added benefit of obviating a dedicated microfluidic sorter with its associated fluorescence detection hardware and complexity. In addition to increasing target enrichment, higher MESA throughput can also increase the amount of target DNA available for downstream sequencing. When combined with new sequencing technologies, MESA should facilitate deep sequencing of long target regions without amplification bias and artifacts, more readily uncovering genetic variations relevant to human disease that are challenging to identify.

The MESA method is, in its most fundamental form, a sorter of nucleic acids and is not limited to enriching target regions within complex genomes. It could also be used to identify and sort RNA, viruses or even uncultivable microbial genomes from mixed populations (44). This approach should prove particularly valuable for facilitating metagenomic studies where limited sequence information is available for probe design *a priori*. MESA might also enable the enrichment of targets from other populations of mixed nucleic acids, such as cell-free DNA or exosomes from blood. We therefore anticipate that MESA, due to its unique ability to capture long molecules using minimal sequence information, will provide a general, new sequence enrichment capability that will prove useful in a variety of applications, especially studies of genetic variation.

## SUPPLEMENTARY DATA

Supplementary Data are available at NAR Online.

SUPPLEMENTARY DATA
