# Supplementary material for: Microfluidic droplet enrichment for targeted sequencing
Source: Nucleic Acids Res. 2015 Apr 14;43(13):e86. doi: 10.1093/nar/gkv297 (PMC4513844; doi:10.1093/nar/gkv297)
Supplement: SUPPLEMENTARY DATA [file supp_43_13_e86__index.html]

Microfluidic droplet enrichment for targeted sequencing — Microfluidic droplet enrichment for targeted sequencing — SUPPLEMENTARY DATA 

# Microfluidic droplet enrichment for targeted sequencing

## SUPPLEMENTARY DATA

**Files in this Data Supplement:**

- SUPPLEMENTARY DATA
