# Supplementary material for: Microfluidic droplet enrichment for targeted sequencing
Source: Nucleic Acids Res. 2015 Apr 14;43(13):e86. doi: 10.1093/nar/gkv297 (PMC4513844; doi:10.1093/nar/gkv297)
Supplement: SUPPLEMENTARY DATA [file supp_gkv297_nar-00454-met-k-2015-File007.pdf]

SUPPLEMENTARY DATA

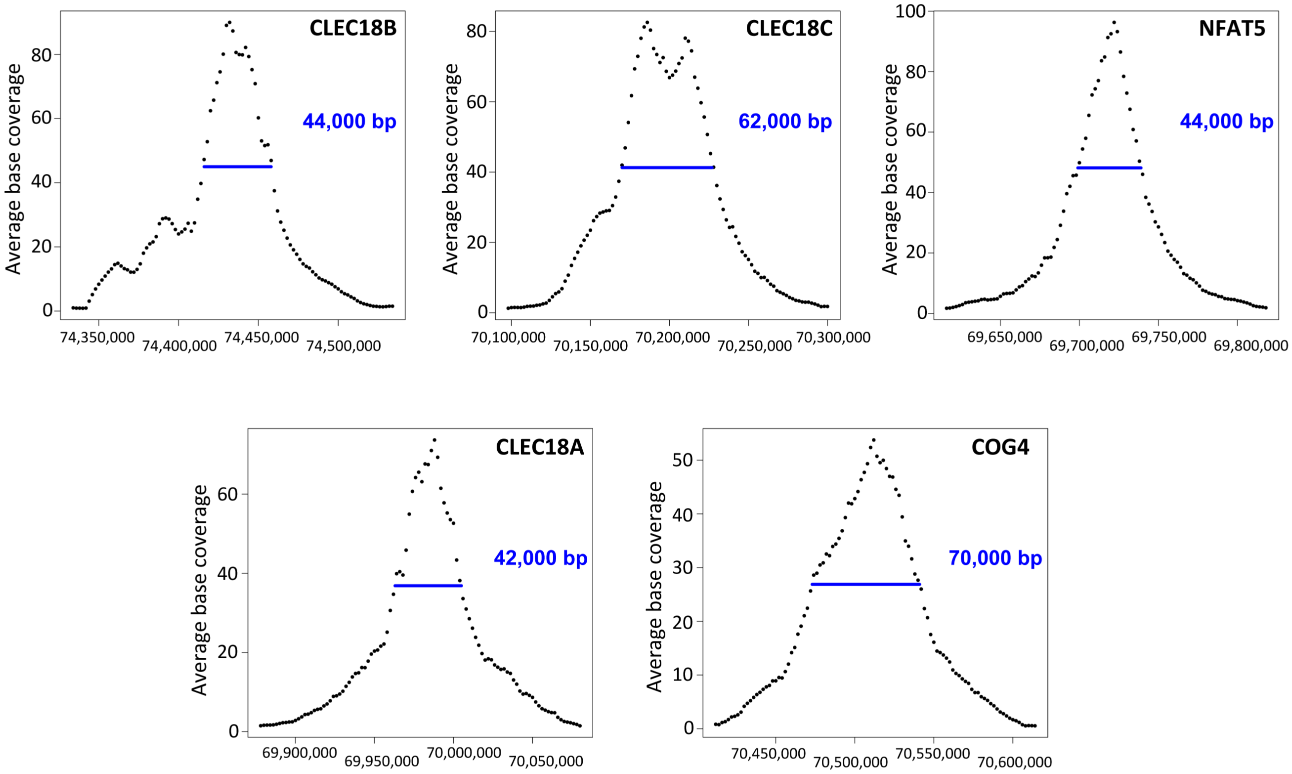

Supplementary Figure S1. *Estimate of the average genomic DNA fragment size encapsulated with the MESA workflow.* 130 million reads were used to calculate the average base coverage in the targeted regions, using a 20 kb sliding window. Each plot (black dots) represents the mean-subtracted coverage distribution of the targeted loci. The full width at half maximum for each peak (blue line) was used to calculate the mean DNA fragment size encapsulated in droplets (52,400 +/- 5,706 bp). The x-axis of each plot indicates the position on human chromosome 16.
